# Analysis of co-expression gene network associated with intracranial aneurysm and type 2 diabetes mellitus

**DOI:** 10.3389/fneur.2022.1032038

**Published:** 2022-12-06

**Authors:** Tian Tian, Wenhao Sun, Jia Du, Yafei Sun

**Affiliations:** ^1^Department of Neurological Surgery, Chengde Medical University Affiliated Hospital, Chengde, China; ^2^Department of Neurological Surgery, Cangzhou Center Hospital, Cangzhou, China; ^3^Department of Neurology, The Second Hospital of Hebei Medical University, Shijiazhuang, China

**Keywords:** intracranial aneurysm, T2DM, STAT1, neurovascular markers, ARNTL2

## Abstract

To screen for common target genes in intracranial aneurysms (IA) and type 2 diabetes mellitus (T2DM), construct a common transcriptional regulatory network to predict clusters of candidate genes involved in the pathogenesis of T2DM and IA, and identify the common neurovascular markers and pathways in T2DM causing IA. Microarray datasets (GSE55650, GSE25462, GSE26969, GSE75436, and GSE13353) from the GEO database were analyzed in this research. Screening of the IA and the T2DM datasets yielded a total of 126 DEGs, among which 78 were upregulated and 138 were downregulated. Functional enrichment analysis revealed that these DEGs were enriched for a total of 68 GO pathways, including extracellular matrix composition, coagulation regulation, hemostasis regulation, and collagen fiber composition pathways. We also constructed transcriptional regulatory networks, and identified key transcription factors involved in both the conditions. Univariate logistic regression analysis showed that ARNTL2 and STAT1 were significantly associated with the development of T2DM and IA, acting as the common neurovascular markers for both the diseases. In cellular experiments, hyperglycemic microenvironments exhibited upregulated STAT1 expression. STAT1 may be involved in the pathogenesis of IA in T2DM patients. Being the common neurovascular markers, STAT1 may acts as novel therapeutic targets for the treatment of IA and T2DM.

## Introduction

Diabetes mellitus (DM) is a clinical syndrome characterized by high blood glucose levels due to a combination of genetic and environmental factors. It is categorized as type 1 (T1DM) or type 2 (T2DM) depending on the underlying cause. T2DM is the more common type of DM, and it is primarily manifested by insulin resistance or relatively insufficient insulin secretion (1). Furthermore, T2DM is usually accompanied by micro and macroangiopathy, including diabetic eye disease, diabetic nephropathy, diabetic foot, cerebral infarction, myocardial infarction, and other manifestations, seriously affecting the quality of life and life expectancy of patients (2–4). However, effective intervention in reducing blood glucose reduces the incidence of cardiovascular events. Daqing et al. found that a 6-year lifestyle intervention reduced the incidence of diabetes and cardiovascular events (heart attack, cerebral infarction, and heart failure) by 49 and 26% over 30 years, in people with impaired glucose tolerance (5).

The molecular mechanisms associated with the development of aneurysms are complex. Although the cause of intracranial aneurysms (IA) is still poorly understood, it is thought to be a result of a combination of factors, including hemodynamic, morphological, and clinical factors (6–8). Hemodynamic disorders can induce endothelial dysfunction, induce macrophages to release pro-inflammatory cytokines and matrix metalloproteinases (MMPs) to digest the extracellular matrix (ECM), and induce apoptosis of vascular smooth muscle cells (SMCs), resulting in the loss of vessel wall integrity and development of aneurysm (9–11). In addition, vascular morphological factors are also important in the development of aneurysm. High glucose-induced reactive oxygen species (ROS) may be involved in the activation of c-Jun N-terminal kinase (JNK) pathway, which in turn triggers caspase-3 and promotes apoptosis of vascular endothelial cells, leading to changes in the vascular structure (12). Additionally, ROS can induce vascular SMC senescence and aneurysm formation by activating nuclear factor kappa-light-chain-enhancer of activated B cells (NF-κB) (13). In contrast, immunosuppressants can limit aneurysm growth by reducing JNK activation, decreasing the inflammatory response, and reducing endothelial cell activation (14).

These studies suggest that T2DM can induce the development of IA; however, the underlying mechanism is unclear. The hemodynamic and vascular structural damage induced by T2DM is considered to be an important factor in the development of aneurysms. Free fatty acid (FA) concentrations are elevated in the blood of diabetic patients due to excessive release of adipose tissue and its reduced uptake by skeletal muscles (15–17). The liver increases very low density lipoproteins (VLDL) production and cholesteryl esters synthesis to eliminate the excess FA. Free cholesterol contributes to atherosclerosis by activating toll-like receptor proteins (TLRs) and prolonging the activation of p38 mitogen-activated protein kinase (MAPK), thereby causing degenerative variations in the arterial wall and promoting the development of aneurysms (18, 19). A few studies have shown that platelet reactivity is elevated in T2DM patients, leading to impaired coagulation regulation, increasing the risk of cardiovascular events (20). In addition, an increase in plasma coagulation factors (e.g., factor VII and thrombin) and a decrease in endogenous anticoagulants (e.g., thrombomodulin and protein C) in DM patients, increases the risk of thrombosis, which can cause altered hemodynamics and aneurysm development (21, 22). A decrease in the expression of MMP-2 and 3 and an increase in the expression of tissue inhibitor of metalloproteinases (TIMP), in response to high glucose induction, can cause vascular lesions that induce aneurysm formation (23).

In previous studies, diabetes and IA prevalence and growth were paradoxically negatively associated, and this protective effect may have been attributed to diabetes drugs (24). As a result, the specific relationship between diabetes and IA remains unclear (25). Diabetes is a risk factor for vascular dysfunction (26). It is a consequence of vascular dysfunction that IA occurs (27, 28). Recent developments in bioinformatics have provided us with an effective method for establishing the link between diabetes and neurovascular diseases, including IA (29–36). Transcription factors (TFs) are an important breakthrough in the study of disease associations (37–40). Currently, it is unclear whether TFs in peripheral blood can serve as effective markers for T2DM and IA. Therefore, identifying TFs that are differentially expressed in peripheral blood of T2DM and IA patients may provide a new avenue for the prevention and diagnosis of T2DM and IA.

In order to further explore the association between T2DM and IA, we analyzed the correlation between the differentially expressed genes (DEGs) common to IA and T2DM through multi-omics analyses and constructed a transcriptional regulatory network by using logistic regression curve analysis. The results of this analysis provide a theoretical basis for IA pathogenesis in T2DM patients and provide theoretical support for its diagnosis and treatment.

## Methods

### Data acquisition and pre-processing

For the following keywords: diabetes mellitus, type 2 diabetes mellitus, or intracranial aneurysms, we searched the NCBI GEO database (https://www.ncbi.nlm.nih.gov/geo/). In order to screen the dataset and ensure that relevant data was recorded, the following criteria were used: (i) samples included both normal and disease samples; (ii) the dataset was capable of completing expression profiling based on the array method; (iii) the species was restricted to Homo sapiens; and (iv) raw data was available for analysis. The IA-associated gene expression microarray datasets (GSE26969, GSE75436, and GSE13353) and T2DM-associated gene expression microarray datasets (GSE55650 and GSE25462) were obtained *via* the Gene Expression Omnibus (GEO) database. GSE26969 dataset included three unruptured IA and the corresponding control samples, GSE75436 included 15 IA and the corresponding superficial temporal artery samples, and GSE13353 included eight unruptured IA samples. The GSE55650 dataset included 12 T2DM and 11 control samples with family history of DM, while the GSE25462 dataset included 10 T2DM and 25 control samples with family history of DM. Thus, we included a total of 26 disease and 18 control samples for IA and 22 disease and 36 control samples for T2DM as show in Table 1.

**Table 1:** Sources of data related to intracranial aneurysms and T2DM.

**ID**	**GSE number**	**Platform**	**Samples**	**Diseases**
1	GSE26969	GPL570	3 patients, 3 controls	Intracranial arterial aneurysm
2	GSE75436	GPL570	15 patients, 15 controls	Intracranial arterial aneurysm
3	GSE13353	GPL570	8 patients	Intracranial arterial aneurysm
4	GSE55650	GPL570	12 patients, 11 controls	Type 2 diabetes
5	GSE25462	GPL570	10 patients, 25 controls	Type 2 diabetes

### Differential expression analysis

R software (version 4.0.2) was used to process the download matrix file and platform. Gene symbols were assigned to the probe names corresponding to the IDs. The mean value was chosen when multiple probes corresponded to one gene. In addition, for each of the two disease categories, we used the ComBat function in the sva (v3.40.0) package to eliminate batch effects between multiple datasets and retain the biological differences between the disease and control samples. Thereafter, we normalized the samples using the normalize Between Arrays function in the limma (v3.48.3) package (41). Differential expression analysis (DEA) was done individually for the IA and the T2DM datasets using the limma package, and the thresholds for DEGs were set as |log2FC| > 1 and FDR <0.05. Furthermore, the intersection analysis is integrated in the dataset using Venn Analytics (Venn).

### Weighted co-expression network construction and module identification

By analyzing gene expression data, WGCNA (Weighted Correlation Network Analysis) constructs gene co-expression networks (42). By analyzing association relationships between genes, WGCNA categorizes genes into modules. Finally, correlation analysis between these modules and sample phenotypes is used to examine molecular features of specific phenotypes. The expression profiles of the two disease categories were integrated and the expression distributions of all the samples were normalized using the normalizeBetweenArrays function in the limma (v 3.48.3), and the genes in the top 25% of the standard deviation (*N*_gene_ = 5,720) were selected for subsequent analysis. Thereafter, we conducted weighted gene co-expression network analysis (WCGNA) using the WGCNA (v 1.70-3) package (43), and the strength of association between the nodes was determined by using the adjacency matrix. The pickSoftThreshold function of WGCNA was used to calculate the scale free fit index (*R*^2^) corresponding to different soft thresholds, and the first soft threshold that marked the *R*^2^ > 0.9 was selected. The soft threshold β was set at 8. Subsequently, we transformed the adjacency matrix into a topological overlap matrix (TOM), to quantitatively describe the similarity of nodes by comparing the weighted correlation between two nodes and other nodes. Thereafter, we performed hierarchical clustering to identify co-expression modules, each containing at least 50 genes. Lastly, we computed module eigengene (ME) and merged the similar modules (abline = 0.1).

### Identification of significant co-expressed genes

Pearson correlations between ME and the disease types were calculated and their significance was calculated using the corPvalueStudent function. Finally, the intersections between the gene modules that were significantly associated with both the disease types and the common DEGs of the two disease types were taken as the target genes.

### Construction of transcriptional regulatory networks

We obtained 8,427 human-related transcriptional regulatory relationships, including 795 TFs from the TRRUST (v2) database, which collects a large number of manually-calibrated TF-target regulatory interactions (44). Subsequently, we extracted the transcriptional regulatory networks associated with the target genes and visualized them using cytoscape (v 3.8.0).

### Enrichment analyses

The enrichGO function in the clusterProfiler (v 4.2.2) package was used to enrich the set of genes of interest into GO entries, and those with a *p*-value of <0.05 were considered to be significantly enriched (45). The samples were divided into high and low expression groups based on STAT1 expression values. Then the GSEA function in the clusterProfiler package was used to screen for activated (*p*-value <0.05 and NES > 1) or inhibited (*p*-value <0.05 and NES <1) biological process (BP) pathways in the different groups.

### Western blot and cell culture for detecting protein expression

The Shanghai Institute of Cell Biology, Chinese Academy of Sciences has provided us with human umbilical vein endothelial cell line (ECV-304). Cells were cultured in RPMI1640 medium containing 10% FBS at 37°C in a 5% CO_2_ incubator. The cells were cultured in groups and given different concentrations of D-glucose to stimulate ECV-304 cell line for the same time (24 h), and the experiment was divided into two groups as follows: (i) control group: Glucose 5.5 mmol/L; (ii) diabetic group: Glucose 16.5 mmol/L. By using the BCA method, total protein was extracted from the cells and quantified. SDS-PAGE electrophoresis of 12% proteins was used for separation, PVDF membranes were sealed with 5% skimmed milk powder for 1 h at room temperature, rinsed three times with TBST, incubated overnight at 4°C with the primary antibody, rinsed with TBST three times, then incubated at room temperature for 1 h with the HRP-labeled secondary antibody, rinsed three times, and then ECL-illuminated for color development. The results were analyzed by chemiluminescence imaging system.

### ComPPI constructs protein interaction networks

The ComPPI database (version 2.1.1) is a comprehensive, open source database for analyzing experimental results in biochemistry, molecular biology, proteomics, as well as proteomic and interactomic research in bioinformatics and network science, contributing to cell biology, medicine, and drug design. STAT1 is the input gene and the result file of ComPPI is exported. R software imports the program file along with the input data file and annotates the file after defining the input parameters. Based on ComPPI, we obtained the STAT1 protein interactions network map.

### Statistical analysis

Each experiment was replicated at least three times. Means and standard deviations (SDs) are used to express quantitative data. All statistical tests and graphs were generated using the Project R software (version 4.0.2). To detect differentially expressed genes (DEGs), Bayesian tests were used. P-values <0.05 were considered significant.

## Results

### Identification of genes co-expressed in IA and T2DM

We selected the GSE26969, GSE75436, GSE13353, GSE55650, and GSE25462 datasets from the GEO database for the analysis to include as many disease samples as possible, while minimizing the impact of batch effects on the analysis. The number of samples, disease types, and platform information included in each dataset are shown in Table 1.

**Figure 1:**
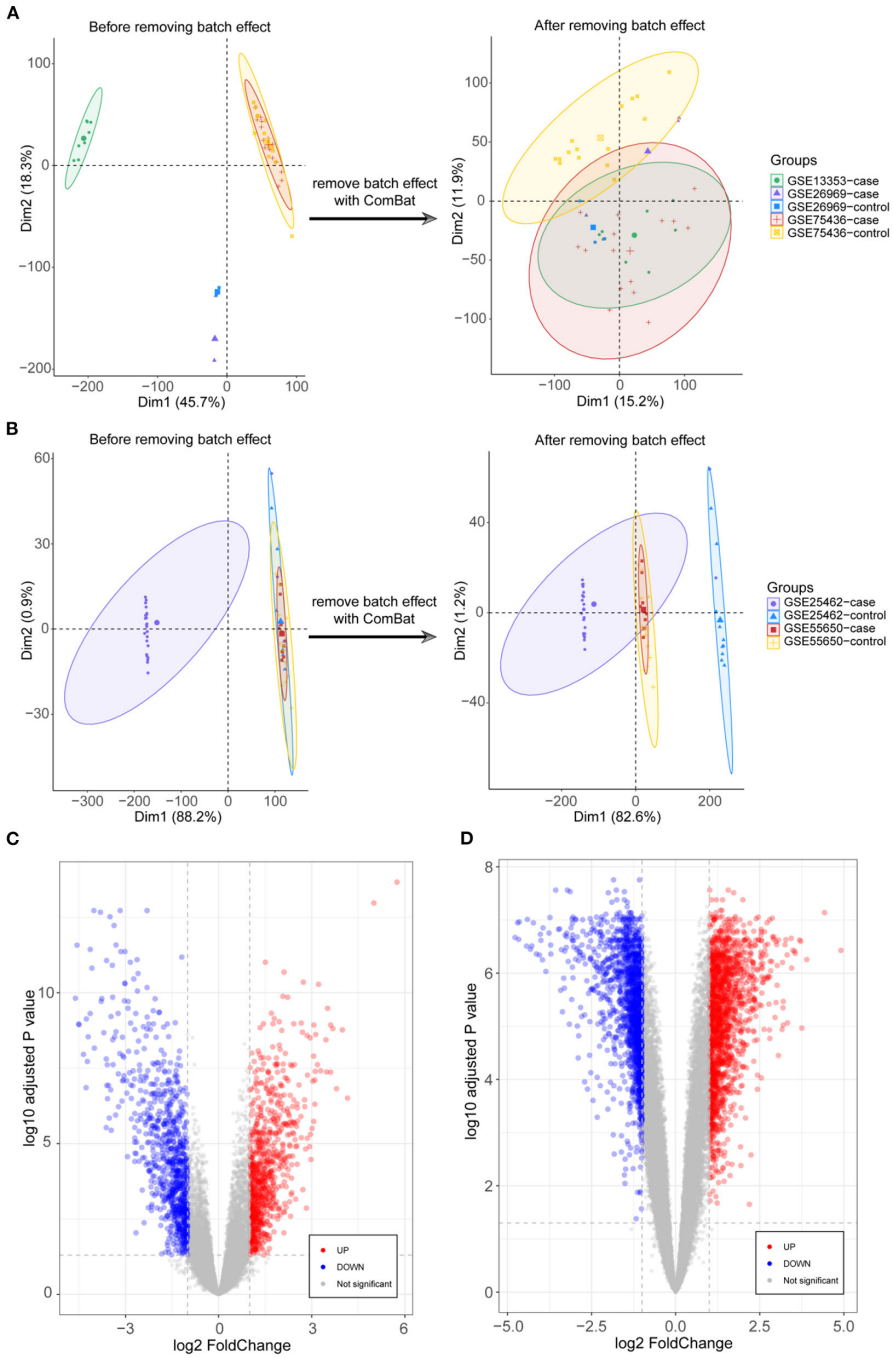
Integration of datasets and differential expression analysis. **(A)** First and second principal components of the intracranial aneurysm (IA)—GSE13353, GSE26969, and GSE75436 datasets before and after eliminating the batch effects. **(B)** First and second principal components of the type 2 diabetes mellitus (T2DM)—GSE25462 and GSE55650 datasets before and after eliminating the batch effects. **(C)** Volcano plots of differentially expressed genes (DEGs) in IA and T2DM. **(D)** Volcano plots of DEGs in T2DM.

DEGs were identified after removing batch effects between multiple datasets (Figures 1A,B). We screened 822 upregulated and 837 downregulated genes in IA and 1678 upregulated and 1474 downregulated genes in T2DM (Figures 1C,D).

### Screening for target genes

**Figure 2:**
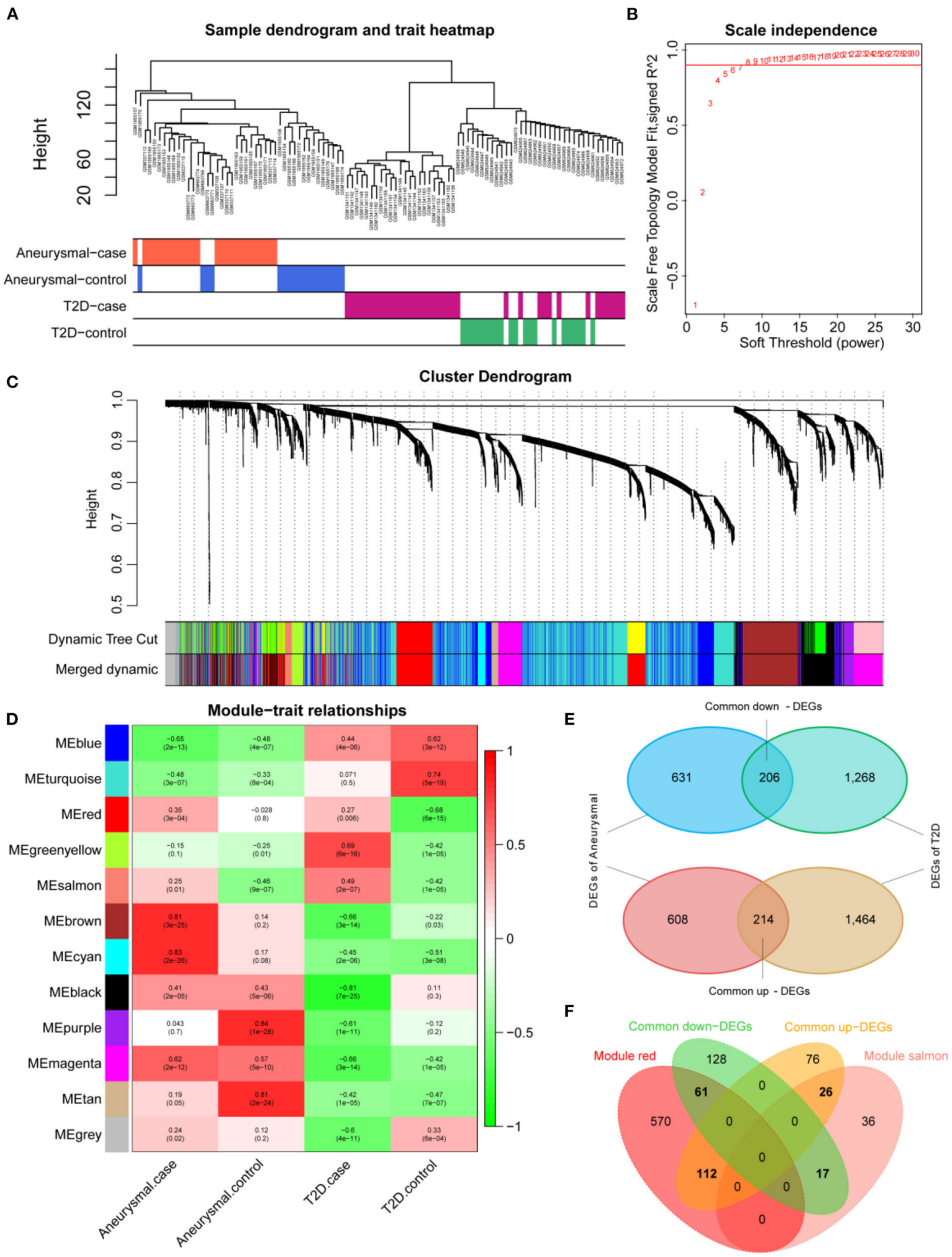
Screening of target genes. **(A)** Dendrogram of clusters for all the samples. **(B)** *R*^2^ of scale-free model fit analysis corresponding to different soft threshold values; *R*^2^ of the red horizontal line is 0.9. **(C)** Dendrogram of the genes in the top 25% of the standard deviation based on dissimilarity measure (1-TOM) clustering; color bars are used to mark different modules. **(D)** Heat map of correlation between module eigengenes (ME) and clinical phenotypes; red and salmon modules are significantly correlated with both intracranial aneurysm (IA) and type 2 diabetes mellitus (T2DM). **(E)** Venn diagram of differentially expressed genes (DEGs) for IA and T2DM. **(F)** Venn diagram of common DEGs for IA and T2DM with red and salmon modules, in which 216 genes were considered as significant target genes.

**Figure 3:**
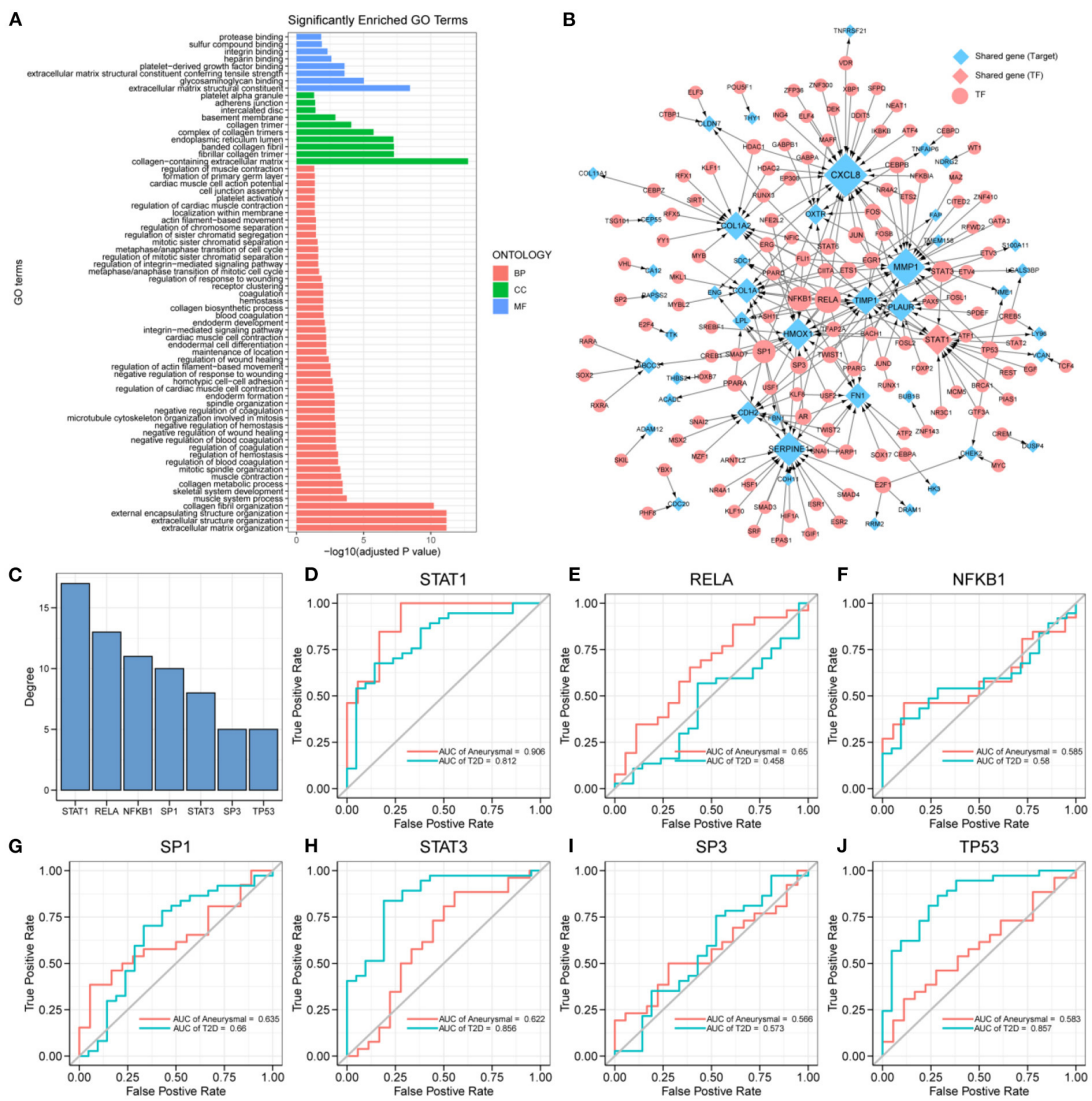
Key transcription factors associated with the target genes. **(A)** 216 target genes enriched to gene ontology (GO) pathways, including 8 molecular function (MF) pathways, 10 cellular component (CC) pathways, and 50 biological process (BP) pathways. **(B)** Transcriptional regulatory networks associated with target genes; the size of nodes is proportional to degree. **(C)** Transcription factors (TFs) with degree ≥5, including: STAT1, RELA, NFKB1, SP1, STAT3, SP3, and TP53. **(D–J)** ROC curves of STAT1, RELA, NFKB1, SP1, STAT3, SP3, and TP53 in intracranial aneurysm (IA) and type 2 diabetes mellitus (T2DM) diagnosis.

The expression profiles of IA and T2DM were integrated and normalized, and hierarchical clustering analyses of all the samples revealed that the different types of samples could be well distinguished (Figure 2A). We used WGCNA to identify the gene modules associated with disease types, and set the soft threshold (β) at 8 for constructing the scale-free network (Figure 2B). Subsequently, we constructed the adjacency matrix and TOM to identify the co-expressed gene modules. A total of 12 gene modules were identified using mean hierarchical clustering and dynamic tree cropping methods (Figure 2C). The red and salmon modules were significantly associated with both IA and T2DM (Figure 2D) and were considered to be clinically significant for subsequent analysis. DEA demonstrated that, in both the diseases, 206 and 214 genes were downregulated and upregulated, respectively (Figure 2E). Furthermore, combined results of DEA and WGCNA revealed that 78 genes were downregulated and 138 genes were upregulated in both the diseases (Figure 2F). These 216 genes showed the same dysregulation pattern in both the diseases and were significantly associated with disease onset, suggesting a possible common pathogenic molecular mechanism between both the diseases; therefore, they were considered as the target genes for IA and T2DM. The target genes were significantly enriched to 68 GO pathways (Figure 3A), including ECM composition, coagulation regulation, hemostasis regulation, and collagen protofibril composition pathways.

### Construction of target genes-related transcriptional regulatory networks and identification of key TFs

The differential expression of target genes in IA and T2DM may be related to the regulation of TFs. To identify the key TFs associated with the occurrence of IA and T2DM, we constructed a target genes-related transcriptional regulatory network (Figure 3B), including 130 TFs and 44 target genes, among which ARNTL2 and STAT1 were both the target genes as well as TFs. Seven TFs in the network had a degree value ≥5 (Figure 3C), and these TFs may play a key regulatory role in the pathogenic mechanism of both the diseases. We further explored the potential clinical diagnostic value of these seven TFs (STAT1, RELA, NFKB1, SP1, STAT3, SP3, and TP53) by plotting their ROC curves based on univariate logistic regression in the diagnosis of the two diseases (Figures 3D–J). The results showed that the AUC values of STAT1 in both diseases were >0.8 indicating that it was significantly associated with the occurrence of both the diseases. Determining its biological functions in IA and T2DM will facilitate the understanding of the common pathogenic mechanisms between IA and T2DM. As a result, STAT1 was selected for further functional pathway analysis.

### Functional pathways involved in STAT1 in IA and T2DM

**Figure 4:**
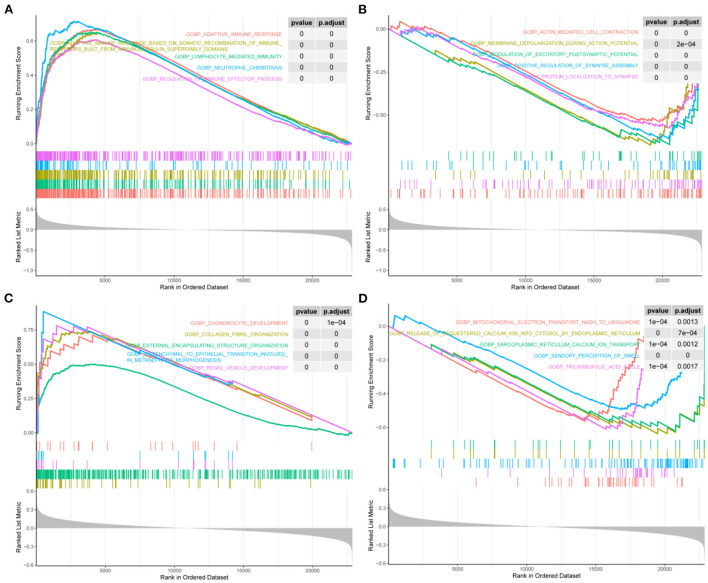
Biological process pathways associated with STAT1 in intracranial aneurysm and type 2 diabetes mellitus. **(A)** Top 5 biological process (BP) pathways (BP) that were significantly activated in patients with intracranial aneurysm (IA) and type 2 diabetes mellitus (T2DM) with high STAT1 expression. **(B)** Top 5 BPs that were significantly inhibited in patients with IA and T2DM with high STAT1 expression. **(C)** Top 5 BPs that were significantly activated in patients with T2DM with high STAT1 expression. **(D)** Top 5 BPs that were significantly inhibited in patients with T2DM with high STAT1 expression.

We screened for the BP pathways associated with STAT1 expression levels in IA and T2DM using gene pooling enrichment analysis. The results revealed that in both IA and T2DM, a large number of immune response-related pathways, including adaptive immune response, lymphocyte-mediated immunity, and neutrophil chemotaxis were activated in the high STAT1 expression group (Figure 4A), while the synaptic-related pathways were inhibited (Figure 4B). In T2DM, the renal vascular development and posterior renal-associated epithelial mesenchymal transition pathways were activated in the high STAT1 expression group (Figure 4C), while the calcium transfer-related pathways were inhibited (Figure 4D). Therefore, STAT1 plays a key role in immune and vascular function-related pathways in IA and T2DM.

### Analysis of diabetes mellitus-related high STAT1 expression and its protein interactions

**Figure 5:**
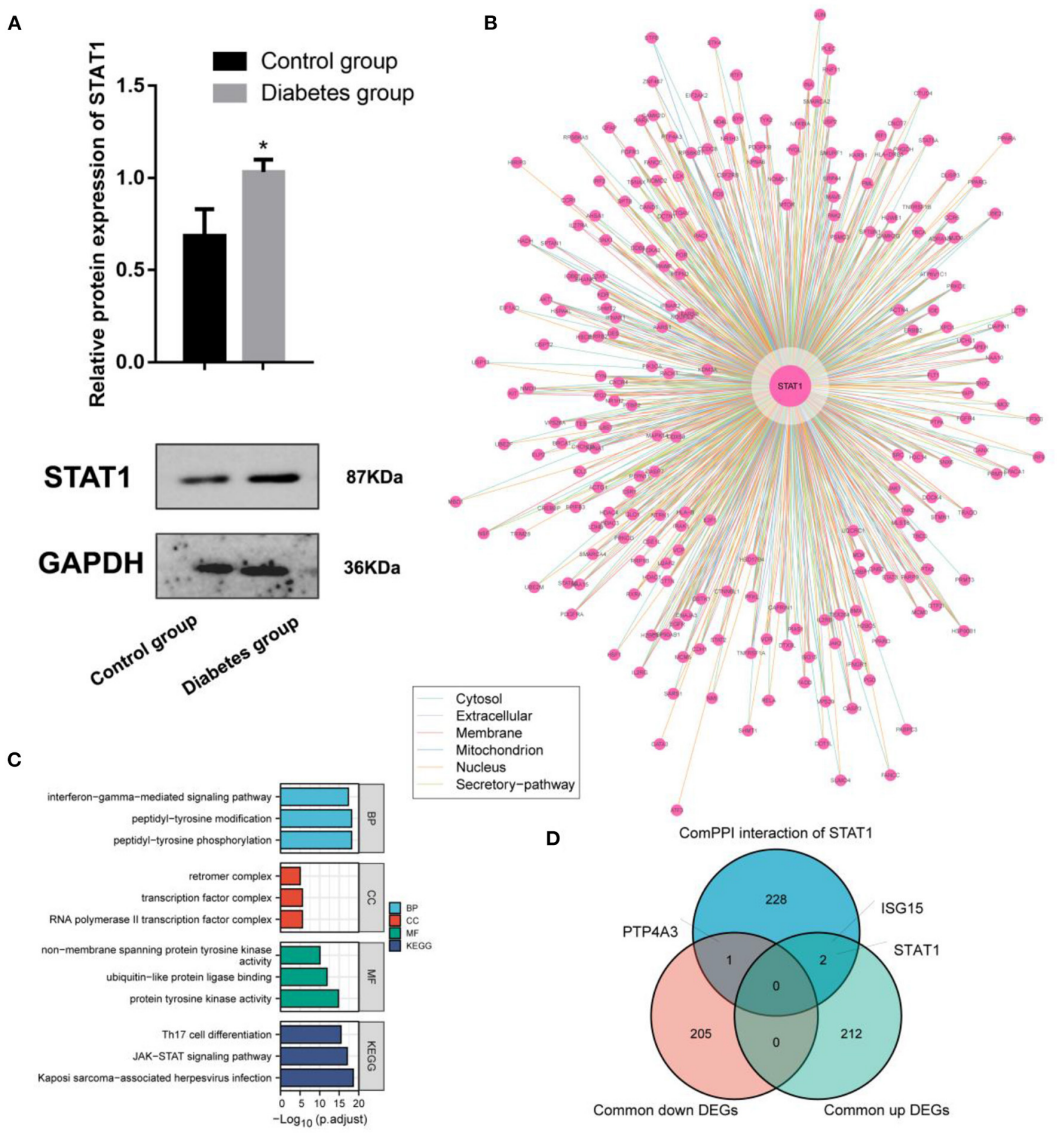
Interaction and functional analysis of the highly expressed STAT1 protein in diabetes mellitus. **(A)** WB experimental study to verify the differential expression of STAT1 in ECV-304 cell lines in the high glucose and control groups; **(B)** Analysis of STAT1 interaction proteins at different cell sites using the ComPPI database; **(C)** Protein interactions with STAT1 enrichment; **(D)** Relationships between proteins interacting with STAT1 and genes differentially expressed in diabetes and IA cross-tabulation analysis. *p < 0.05.

Experimental studies with WB demonstrated that ECV-304 cells exposed to high glucose expressed STAT1 (*p* < 0.05) (Figure 5A). The hyperglycemic microenvironment may upregulate STAT1 in endothelial cells by upregulating STAT1. The ComPPI database was used to identify the proteins that interact with STAT1 at different cell sites (Figure 5B). GO functions enriched in these STAT1-interacting proteins include interferon–gamma–mediated signaling pathway, peptidyl–tyrosine modification, peptidyl–tyrosine phosphorylation, retromer complex, transcription factor complex, RNA polymerase II transcription factor complex, non–membrane spanning protein tyrosine kinase activity, ubiquitin–like protein ligase binding, and protein tyrosine kinase activity (Figure 5C). And the STAT1-interacting proteins are mainly found in the KEGG pathways of Th17 cell differentiation, JAK–STAT signaling pathway, and Kaposi sarcoma–associated herpesvirus infection (Figure 5C). In diabetes and IA, ISG15 was upregulated as a co-interacting protein of STAT1, while PTP4A3 was downregulated as a co-interacting protein of STAT1 (Figure 5D). Accordingly, ISG15 and PTP4A3 may interact with STAT1 to affect diabetes and IA, though more studies are needed (Table 2).

**Table 2:** Protein information of PTP4A3 and ISG15 obtained from the ComPPI database.

**Source**	**Target**	**Location**	**ComPPI score**	**Expression in diabetes and IA**
STAT1	PTP4A3	Cytosol/mitochondrion/nucleus/secretory-pathway/extracellular	0.9974	Common down
	ISG15	Cytosol/nucleus/extracellular/extracellular	0.9600	Common up

## Discussions

T2DM accounts >90% of DM cases globally, and it is characterized by hyperglycemia, low insulin production, and insulin resistance. Long-term hyperglycemia is likely to lead to poor blood flow to the extremities, resulting in reduced vascular elasticity and blood flow blockage. IA are abnormal bulges that occur in the walls of intracranial arteries. Although its etiology is unclear, it is believed that cerebral arteriosclerosis and rising vascular pressure are related to its development. Comparative analysis of the symptoms and causative factors of both the diseases suggested the existence of a common pathogenesis. Therefore, we used RNA-seq data from public databases to obtain genetic features as well as regulatory mechanisms that are common between IA and T2DM. In this study, a total of 216 DEGs were screened from 5 GSE datasets, among which 78 were upregulated and 138 were downregulated. Additionally, 12 gene modules were identified using WGCNA, two of which were significantly associated with both IA and T2DM. Furthermore, in the transcriptional regulatory network constructed using the DEGs, ARNTL2 and STAT1 were identified to be the target genes as well as TFs in both the disease samples.

ARNTL2, also known as BMAL2, belongs to the PAS (Per-Arnt-Sim) superfamily. PAS proteins play an important role in adapting to the circadian oscillations, and disruption of circadian rhythms leads to predisposition to metabolic syndromes, such as obesity and diabetes (46–48). A cohort study showed that the A/G and A/A genotypes of BMAL2 rs7958822 showed a higher adjusted advantage ratio than the G/G genotype in obese men (OR = 2.2), suggesting a significant association between the BMAL2 rs7958822 genotype and T2DM in obese subjects (49). BAML2 can regulate circadian rhythms, and interventions in the circadian patterns of activity and feeding can have significant effects on body weight and metabolism (47, 50). A study revealed that insulin resistance and blood glucose concentrations were improved after overexpression of BMAL2 (51).

STAT1 belongs to the STAT family and mediates the expression of a variety of genes (52–54). Several studies have reported that STAT1 gain-of-function mutations induce the diabetes and multiple autoimmune diseases (55–57). Furthermore, it has been shown that diabetes risk factors, such as hyperglycemia and hyperlipidemia can exacerbate diabetes symptoms by activating NF-κB and STAT1, which together reduce the number of B cells. Another study indicated that CD40L, the physiological ligand of TNFR-5, can activate NF-κB activity in pancreatic islet β-cells, thus inducing islet cell death (58). In addition, one study found that inhibition of the JAK1-STAT1 pathway could protect pancreatic β-cells from cytokine-induced cell death, and improving the T2DM symptoms (59). STAT1 can also be involved in interferon-γ (IFN-γ), TLR4, and interleukin-6 (IL-6) activation pathways thereby amplifying pro-inflammatory signals, leading to increased SMC leukocyte migration, leukocyte adhesion to endothelial cells, and foam cell formation, thereby promoting atherosclerosis and atheroma formation (60).

Through the enrichment analysis of ARNTL2 and STAT1, target genes were enriched to 68 GO pathways, including ECM composition, coagulation regulation, hemostasis regulation, and collagen fiber composition pathways. Among these, BMAL2 regulates the transcription of anticoagulant thrombomodulin and PAI-1 by forming a heterodimer with CLOCK; therefore, abnormal expression of BMAL2 may cause coagulation disorders (61, 62). STAT1 inhibits thrombin-induced STAT-DNA binding activity and TIMP-1 mRNA expression, thereby inhibiting the coagulation process and promoting thrombosis (63). Furthermore, thrombosis can lead to hemodynamic changes that may promote the development of aneurysms. Therefore, it may be assumed that BAML2 and STAT1 can influence the development of aneurysmal complications in T2DM.

In summary, we screened ARNTL2 and STAT1 by constructing a transcriptional regulatory network through multi-omics analyses, and the gene pathways were enriched for clotting regulation and ECM-related pathways. Therefore, it is important to investigate their role in the hemostatic regulatory pathways in T2DM, which can help in the diagnosis and treatment of future complications. However, our study is yet to be validated by *in vitro* experiments to further clarify the specific molecular mechanisms.

## Conclusions

ARNTL2 and STAT1 are aberrantly expressed in T2DM and IA and act as common neurovascular markers for both the diseases. ARNTL2 and STAT1 are involved in hyperglycemic metabolism and coagulation-related regulation of T2DM, causing aneurysms. The findings of this study provide novel diagnostic and therapeutic targets for T2DM complications.

## Data source

In the paper, microarray datasets (GSE55650, GSE25462, GSE26969, GSE75436, and GSE13353) from the GEO database were analyzed.

## Data availability statement

The original contributions presented in the study are included in the article/supplementary material, further inquiries can be directed to the corresponding author/s.

## Author contributions

TT and YS conceived and designed the analysis. TT, WS, and YS collected the data. TT, WS, JD, and YS performed the analysis and wrote the paper. All authors contributed to the article and approved the submitted version.

## Conflict of interest

The authors declare that the research was conducted in the absence of any commercial or financial relationships that could be construed as a potential conflict of interest.

## Publisher's note

All claims expressed in this article are solely those of the authors and do not necessarily represent those of their affiliated organizations, or those of the publisher, the editors and the reviewers. Any product that may be evaluated in this article, or claim that may be made by its manufacturer, is not guaranteed or endorsed by the publisher.
